# Two-Dimensional Micro-/Nanoradian Angle Generator with High Resolution and Repeatability Based on Piezo-Driven Double-Axis Flexure Hinge and Three Capacitive Sensors

**DOI:** 10.3390/s17112672

**Published:** 2017-11-19

**Authors:** Xinran Tan, Fan Zhu, Chao Wang, Yang Yu, Jian Shi, Xue Qi, Feng Yuan, Jiubin Tan

**Affiliations:** Center of Ultra-precision Optoelectronic Instrument Engineering, Harbin Institute of Technology, Harbin 150080, China; tanxr@hit.edu.cn (X.T.); Wangchao.hit@gmail.com (C.W.); yuyang9412@gmail.com (Y.Y.); hitshijian@gmail.com (J.S.); hgdqixue@gmail.com (X.Q.); yuanf@hit.edu.cn (F.Y.)

**Keywords:** angle generator, angular deflection, angle calibration, flexure hinge, capacitive sensor, Monte Carlo method

## Abstract

This study presents a two-dimensional micro-/nanoradian angle generator (2D-MNAG) that achieves high angular displacement resolution and repeatability using a piezo-driven flexure hinge for two-dimensional deflections and three capacitive sensors for output angle monitoring and feedback control. The principal error of the capacitive sensor for precision microangle measurement is analyzed and compensated for; so as to achieve a high angle output resolution of 10 nrad (0.002 arcsec) and positioning repeatability of 120 nrad (0.024 arcsec) over a large angular range of ±4363 μrad (±900 arcsec) for the 2D-MNAG. The impact of each error component, together with the synthetic error of the 2D-MNAG after principal error compensation are determined using Monte Carlo simulation for further improvement of the 2D-MNAG.

## 1. Introduction

The angle is one of the most important basic geometrical quantities in the field of precision engineering [1,2,3,4]. Various microangle measurement instruments, such as the angle encoder [5,6,7], autocollimator [8,9], and angle interferometer [10], are commonly used in scientific research and industrial metrology to provide accurate angle reference values within the feedback loop of manufacturing and testing processes.

As angle measurement progresses from micro- to nanoradian scale, the systematic errors of these measurement instruments account for an increasingly significant proportion of the angle measurement results. Thus, calibration processes for determining these systematic errors are particularly important. Ongoing developments in the domain of angle calibration has created a demand for improved angle standardization equipment and microangle generators (MAGs).

An MAG is an important and essential functional unit in angle metrology and calibration, which outputs microangles according to sampling intervals selected in the angle range to be calibrated. If permitted by the testing time and data processing capability, very small sampling intervals can be used, which better reveal the nonlinear errors in both the local and full angular ranges of the microangle measurement instrument. In some special cases, such as applications to determine the interpolation error in an angular range of 47.9 μrad (9.89 arcsec) corresponding to the two adjacent lines of the circular grating in the German Physikalisch-Technische Bundesanstalt (PTB) primary angle standard [5,6,7], or to determine the nonlinear error in an angular period of 11.7 μrad (2.41 arcsec) corresponding to the pixel size of the photoelectric detector in the Elcomat 3000 autocollimator [8,9], the selected angle interval must be sufficiently small to yield sufficient sampling points for error evaluation. In addition, multiple tests are usually needed at each angle interval during the calibration process. The requirements of a small angle interval and multiple tests necessitate MAGs with a high angle output resolution and repeatability.

Current MAGs are mainly based on a structure comprised of a precision rotary table and piezo-driven flexure hinge. However, the manufacture of the rotary table, especially for two-dimensional (2D) angle output, is complex and expensive [11,12,13,14,15]. In recent years, owing to the traceability requirements for the radian SI (Système International d'Unités) unit for plane angles in the fields of precision mechanics, nanopositioning, optical fabrication, etc., a flexure-hinge-based MAG with nanoradian resolution has been developed. For example, in 2009, a flexure-hinge-based sine-bar-type nanoradian angle generator was developed at the Istituto Nazionale di Ricera Metrologica (INRiM), Italy [16]. This generator is driven by PZT (piezoelectric transducer), and its output angle is monitored by a capacitive sensor and a two-mirror-based multireflection microangle amplifier. Hence, a nanoradian angle output resolution and uncertainty of 20 nrad (0.004 arcsec) in a range of 120 μrad (24.8 arcsec) can be achieved. In 2012, a flexural-pivot-bearing-based MAG that can achieve an angle output resolution of 5 nrad (0.001 arcsec) was developed at the Ulusal Metroloji Enstitüsü (UME), Turkey [17]. Further, in 2015, Diamond Light Source Ltd., UK, developed a cartwheel-flexure-based nanoangle generator that can reliably provide 1 nrad (0.0002 arcsec) minimal incremental steps in a range of more than 7000 μrad (1444 arcsec) [18]. Finally, Physik Instrumente (PI), Germany, has developed several products that can output tilt angles in two dimensions using a flexure hinge with a high resolution of 20 nrad (0.004 arcsec) and high repeatability of 60 nrad (0.012 arcsec) in the range of 350 μrad (72.2 arcsec) [19,20,21,22,23].

The above reports indicate that the existing rotary table and flexure-hinge-based MAGs are highly developed and can achieve nanoradian angle output resolution. However, existing MAGs mostly provide one-dimensional (1D) angle output, whereas the high accuracy and repeatability of 2D-MAGs are limited in the case of a large output angle range. In many cases, 2D-MAG angle outputs are needed to satisfy 2D angle calibration requirements, for example, for the typical calibration of an autocollimator.

This study proposes a flexure-hinge-based 2D-MNAG that employs a piezo-driven double-axis flexure hinge for angular deflection and three high-precision capacitive sensors for output angle monitoring and feedback control. The principal error of the capacitive sensor for precision microangle measurement is analyzed and compensated for to guarantee high angle output resolution and positioning repeatability for the 2D-MNAG over a large angle range in 2D. The impact of each error component together with the synthetic error of the 2D-MNAG after principal error compensation are also determined using Monte Carlo simulation for the further improvement of the 2D-MNAG.

## 2. Double-Axis Flexure Hinge Based 2D-MNAG

**Figure 1:**
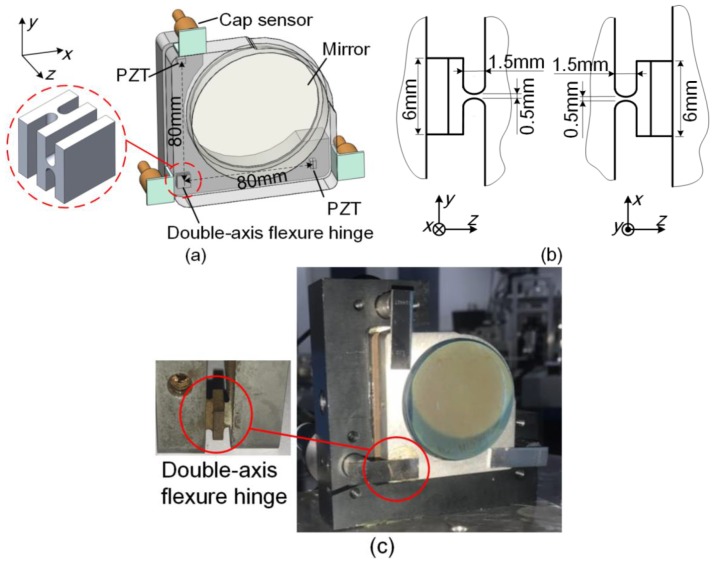
Design of two-dimensional micro-/nanoradian angle generator (2D-MNAG): (**a**) basic structure; (**b**) critical dimension of double-axis flexure hinge; and, (**c**) picture of actual 2D-MNAG.

Figure 1a shows the basic structure of the double-axis flexure-hinge-based 2D-MNAG. The double-axis flexure hinge is constructed using two orthogonally stacked one-axis flexure hinges having zero friction, high motion resolution and accuracy, high positioning repeatability, etc. The two driving points are arranged in an L-shape relative to the bearing point of the double-axis flexure hinge to achieve angular deflection in 2D. Figure 1b shows the critical dimensions of the double-axis flexure hinge.

The basic structure of the 2D-MNAG, including the flexure hinge, is processed from bulk spring steel using wire-electrode cutting. Two PZTs of N-472.110 type with an E-871 controller (PI, Germany) are used to drive the 2D-MNAG, because of their high resolution of less than 1 nm and a large output range of 7.5 mm. Three CS1HP capacitive sensors with a DT6530 controller (Micro Epsilon, U.K.) are used to monitor the 2D-MNAG output angle. Each CS1HP capacitive sensor with a DT6530 controller has a resolution of 0.75 nm and a measurement range of 1 mm. The 2D-MNAG was moved in a closed loop and a proportional-integral-derivative (PID) controller with a non-control zone of ±0.003 arcsec was used.

The dynamics of the 2D-MNAG is limited by both the flexure hinge and the capacitive sensor. The first-order dynamics of the flexure hinge is 79.09 Hz, which is simulated using SolidWorks software. The dynamics of the CS1HP capacitive sensor with DT6530 controller, which is presented in the technical document of the product [24], is 2 Hz during operation at 0.75-nm resolution. It seems that the dynamics of the capacitive sensor must primarily be improved. However, at present, the 2D-MNAG can be used to yield a micro-angle with high precision for a relatively large angle range at low frequency.

Figure 1 shows that the position of the deflection plane is uniquely determined using the three capacitive sensors. Thus, indeterminacy of the bearing point position during the 2D angular deflection of the plane can be avoided. The mathematical relationship between the outputs of the three capacitive sensors and the deflection angle of the plane, that is, the angle output of the 2D-MNAG, is analyzed in detail below.

**Figure 2:**
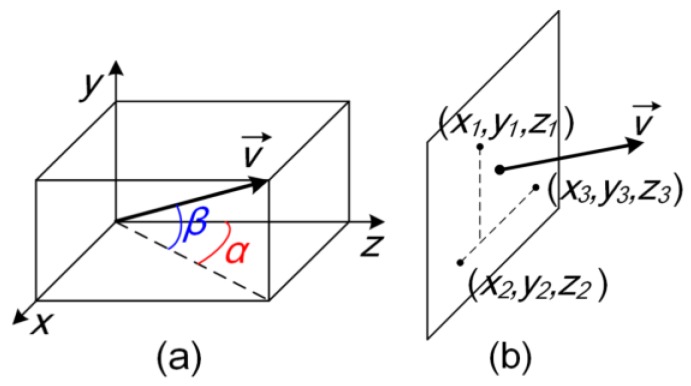
Definition of 2D angle or spatial angle: (**a**) 2D angle and the normal vector; (**b**) Three points and the normal vector.

Figure 2 shows the definition of the 2D angle, which is expressed as (*α*, *β*). The mathematical relationship between the 2D angle and the coordinates of the three monitoring points of the deflection plane are given as follows:

(1) $$\left\{ \begin{array}{l} \alpha =\arctan\frac{\left(y_{2}-y_{1}\right)\left(z_{3}-z_{1}\right)-\left(y_{3}-y_{1}\right)\left(z_{2}-z_{1}\right)}{\left(x_{2}-x_{1}\right)\left(y_{3}-y_{1}\right)-\left(x_{3}-x_{1}\right)\left(y_{2}-y_{1}\right)}\\ \beta =\arcsin\left(\left(x_{3}-x_{1}\right)\left(z_{2}-z_{1}\right)-\left(x_{2}-x_{1}\right)\left(z_{3}-z_{1}\right)\right) \end{array}\right.$$

where (*x*_1_, *y*_1_, *z*_1_), (*x*_2_, *y*_2_, *z*_2_) and (*x*_3_, *y*_3_, *z*_3_) are the coordinates of the three points on the rotation plane, for which (*x*_1_, *y*_1_), (*x*_2_, *y*_2_), and (*x*_3_, *y*_3_) are determined by the mechanical structure while *z*_1_, *z*_2_ and *z*_3_ are monitored by the three capacitive sensors.

According to Equation (1), the desired 2D angle output can be obtained by controlling the *z*-axis positions of the monitoring points read by the three capacitive sensors.

## 3. Capacitive-Sensor-Based 2D Angle Monitoring Unit

### 3.1. Analysis of Principal Error

**Figure 3:**
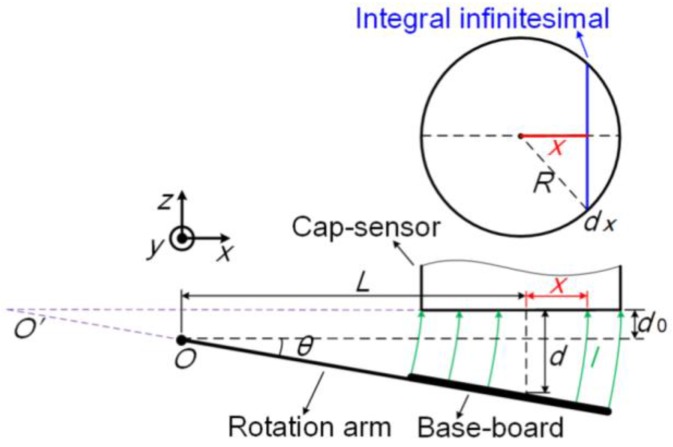
Principal error analysis of capacitive-sensor-based angle monitoring unit.

The capacitive-sensor-based angle monitoring unit operates based on the tangent principle. As shown in Figure 3, the capacitive sensor is fixed while the baseboard is rotated by the rotation arm. The deflection angle can be calculated using the distance monitored in real time by the capacitive sensor, such that:

(2) $$\theta =\arctan\left(\frac{d-d_{0}}{L}\right)$$

where *d*_0_ and *d* are the distances between the capacitive sensor and baseboard before and after angular deflection, respectively, and *L* is the effective length of the rotation arm.

The capacitance between the sensor plane and baseboard is translated into distance in the sensor controller, according to the mathematical relationship for the parallel plate capacitor. However, the sensor plane is not parallel with the baseboard, which is deflected with the rotation arm during the angle measurement. Thus, the distance given by the sensor controller is not exactly that from the center of the sensor plane along its axis to the baseboard. This is the source of the principal error in the capacitive-sensor-based micro-/nanoangle measurement method. This principal error is analyzed in detail below.

The capacitance can be calculated using the integral shown in Equation (3) when the baseboard is deflected by the rotation arm. A detailed derivation of Equation (3) is given in Appendix A.

(3) $$C=\int dC=\int_{-R}^{R}\frac{2\varepsilon_{0}\varepsilon_{r}\sqrt{R^{2}-x^{2}}}{\left(\frac{d}{\tan\theta }+x\right)\theta }dx$$

where *ε*_0_ is the permittivity of vacuum; *ε_r_*, the relative dielectric constant between the sensor plane and baseboard; *R*, the radius of the effective sensor plane; *x*, the distance from the center of the sensor plane to the integral infinitesimal; and, *θ*, the angle between the sensor plane and baseboard.

The capacitance *C* given in Equation (3) is translated to the distance *d_m_* in the sensor controller as follows:

(4) $$d_{m}=\frac{\varepsilon_{0}\varepsilon_{r}\pi R^{2}}{C}$$

where *d_m_* is the measurement value of the distance *d* from the center of the sensor plane along its axis to the baseboard.

Combining Equation (2)–(4), the principal error of the capacitive-sensor-based micro-/nanoangle measurement method, which arises from the nonparallelism of the sensor plane and baseboard, can be calculated using Equation (5). Note that the principal error must be calculated in advance and compensated for in the angle measurement results.

(5) $$Err=\theta_{m}-\theta =\arctan\left(\frac{d_{m}-d_{0}}{L}\right)-\arctan\left(\frac{d-d_{0}}{L}\right)$$

The Micro-Epsilon CS1HP capacitive sensor is taken as an example to show the magnitude of the principal error in the micro-/nanoangle measurement. The initial distance *d*_0_ between the sensor plane and baseboard is 0.5 mm. The effective length *L* of the rotation arm is 100 mm. The CS1HP resolution is 0.75 nm; this predicts a theoretical angle measurement resolution of up to 7.3 nrad (0.0015 arcsec). The angle measurement range is limited to ±4363 μrad (±900 arcsec) as the measurement range of the CS1HP is 1 mm.

**Figure 4:**
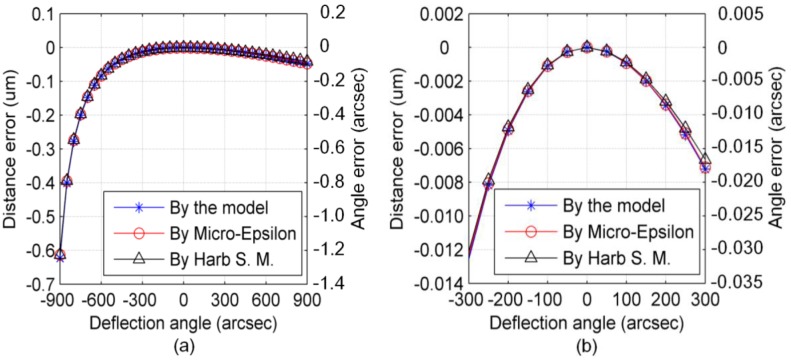
Principal error of capacitive-sensor-based angle measurement method in range of: (**a**) ±900 arcsec; (**b**) ±300 arcsec.

The maximum distance measurement error of the capacitive sensor is ~0.6 μm when the baseboard is deflected by the rotation arm in the angle range of ±4363 μrad (±900 arcsec), yielding an angle measurement error of 6.06 μrad (1.25 arcsec), as shown in Figure 4a. The distance measurement error and leading angle measurement error in the range of ±1454 μrad (±300 arcsec) are 12 nm and 121 nrad (0.025 arcsec), respectively, as shown in Figure 4b.

The simulation results obtained for the established model can be compared to those of the model given by Micro-Epsilon [24] and the model that is given by Harb S. M. in Reference [25,26]. As shown in Figure 4, the results of all the three models are very similar to each other, which can be used for cross-check of the validity of all the three models. However, the models of both Micro-Epsilon and Reference [25,26] are suitable for amending distance measurement results using the known tilt-angle, which cannot be used in angle measurement applications. Thus, the utility of the established model of the capacitive-sensor-based 2D angle monitoring unit presented in this study is verified.

Figure 4a shows that the principal error increase dramatically as the angle output goes to −4363 μrad (−900 arcsec). This is because the distance measurement error tends to be much more sensitive to the angular tilt as the distance between the capacitive sensor and the baseboard is reduced. Therefore, principal error compensation is needed when a capacitive sensor is used for precision angle measurement. The principal error can be well compensated for using Equation (5).

### 3.2. Analysis of Error Sources

A three-dimensional (3D) spatial geometrical model of the angle monitoring unit is established and is used for principal error analysis and the compensation of angle measurement results. Both the sensor plane and baseboard can be expressed using the normal vector and one point of the plane. The rotation of the baseboard can be expressed as the rotation of its point and normal vector around the rotation axis. The measurement value of the baseboard rotation angle is calculated using the formula that is derived in Appendix B.

As the principal error is well compensated for using Equation (5), other factors, such as the machining and assembly error of the mechanical components, and the distance measurement error of the capacitive sensor, become the main factors impacting the microangle measurement.

**Figure 5:**
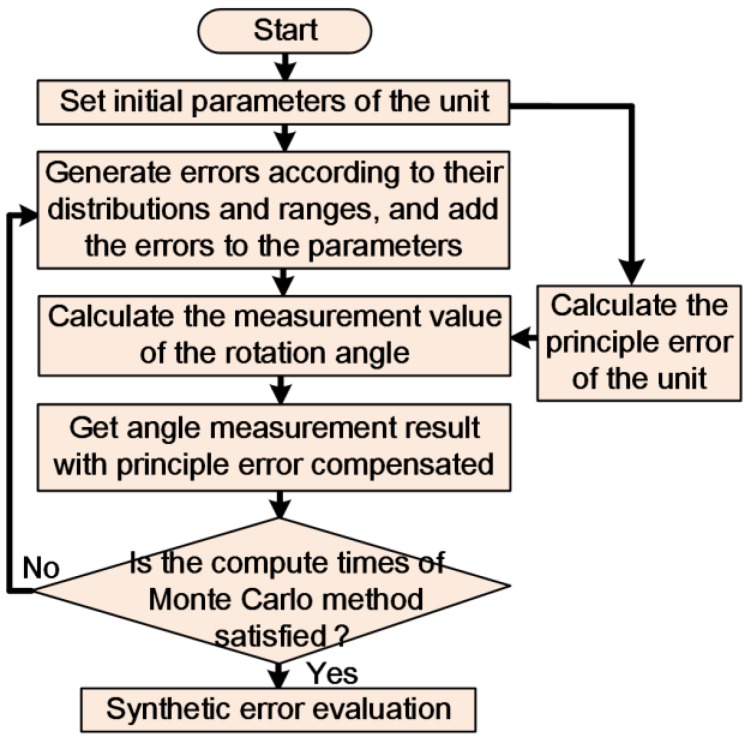
Monte Carlo simulation of capacitive-sensor-based angle monitoring unit.

The Monte Carlo simulation is used to analyze the impact of each error source and the synthetic error of the angle monitoring unit, as shown in Figure 5. The initial distance between the capacitive sensor and baseboard is 0.5 mm, and the effective length of the rotation arm is 100 mm. Table 1 shows the error sources and their impacts on the microangle measurement. The number of computations in the Monte Carlo method is set to 10,000. A simulation is performed to obtain the angle measurement errors and their statistical characteristics at an angle interval of 1454 μrad (300 arcsec) in the range of ±4363 μrad (±900 arcsec).

Because the angle measurement errors are most sensitive to the error sources at the angular position of −4363 μrad (−900 arcsec), the standard deviations of the angle measurement errors at this position are listed in Table 1 to show the impact of each error source. It can be seen that the position error of the center of the capacitive sensor has the maximum impact on the microangle measurement; therefore, this is the primary issue requiring improvement.

**Figure 6:**
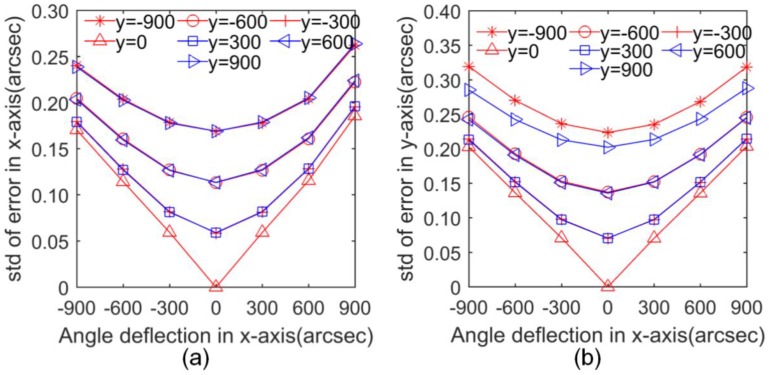
Standard deviations of angle measurement errors of capacitive-sensor-based angle monitoring unit along (**a**) *x*-axis and (**b**) *y*-axis.

**Figure A1:**
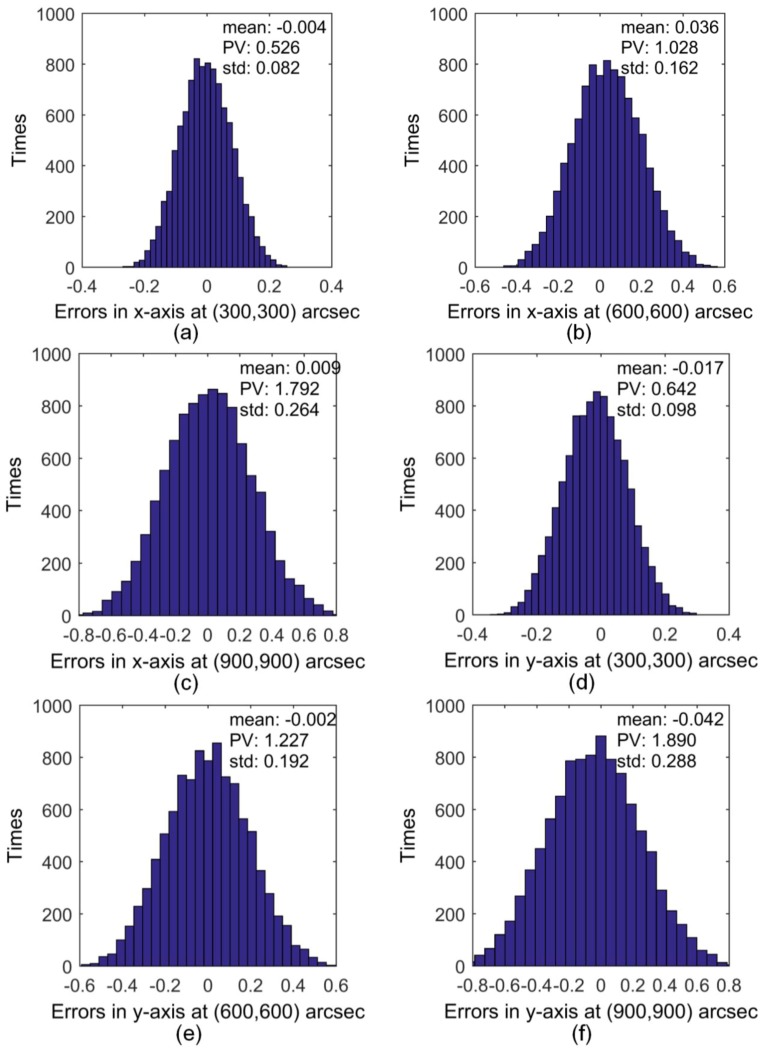
Distribution of angle measurement errors of capacitive-sensor-based angle monitoring unit at different angle positions: (**a**) along *x*-axis at (300, 300) arcsec; (**b**) along *x*-axis at (600, 600) arcsec; (**c**) along *x*-axis at (900, 900) arcsec; (**d**) along *y*-axis at (300, 300) arcsec; (**e**) along *y*-axis at (600, 600) arcsec; (**f**) along *y*-axis at (900, 900) arcsec.

Appendix C (Figure A1) shows part of the distributions of the synthetic errors due to all of the error sources. Figure 6 shows the dispersion of these errors as calculated from the standard deviation at each angle interval of the 2D-MNAG. The maximum standard deviations along the *x*- and *y*-axes are 1280 nrad (0.264 arcsec) and 1547 nrad (0.319 arcsec) in the range of ±4363 μrad (±900 arcsec), respectively, and 398 nrad (0.082 arcsec) and 475 nrad (0.098 arcsec) in the range of ±1454 μrad (±300 arcsec), respectively. The simulation results reveal the microangle measurement property of the capacitive-sensor-based angle monitoring unit when the error sources are controlled within the range that is considered for Table 1. The simulation results can also be used as a reference for relevant designs of other capacitive-sensor-based microangle measurement units.

## 4. Experimental Results

### 4.1. Minimal Angle Increment and Scale Factor Test

**Figure 7:**
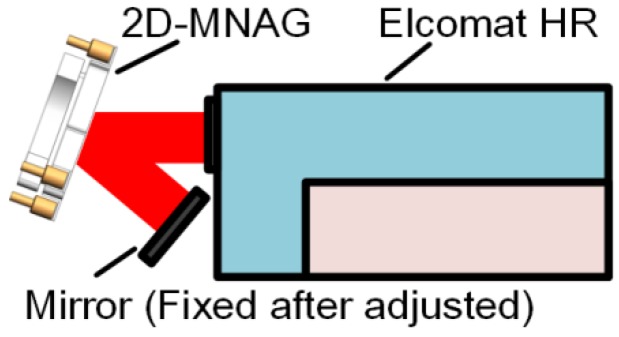
Schematic diagram of test of both minimal angle increment and repeatability of 2D-MNAG.

A piezo driver based on the stick-slip principle is used in the 2D-MNAG to achieve high resolution, large output range, and fast response characteristics. The minimal angle increment of the 2D-MNAG is tested using the setup shown in Figure 7. The output angle of the 2D-MNAG is monitored using both the capacitive-sensor-based angle monitoring unit and an Elcomat HR autocollimator to facilitate a comparison in order to guarantee the reliability of the angle monitoring results. The collimating beam of the Elcomat HR is reflected twice by the 2D-MNAG; thus, the angle measured by the Elcomat HR is double that as measured by the 2D-MNAG, enabling easier and more accurate detection of the minimal angle increment of the 2D-MNAG.

**Figure 8:**
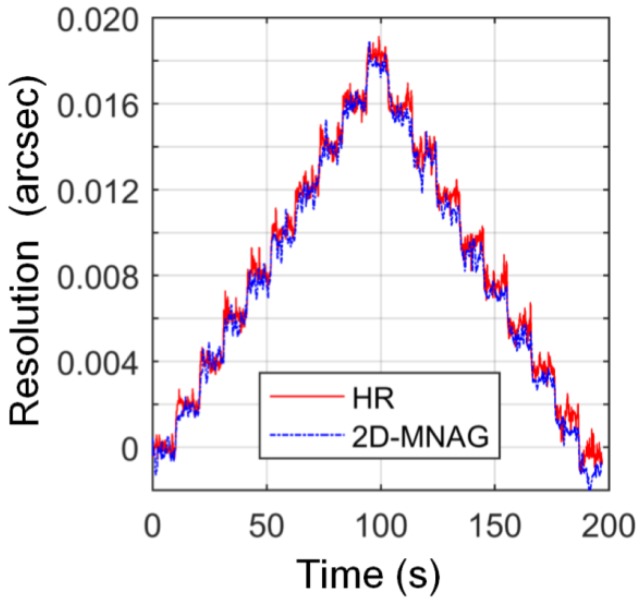
Angle increment of 9.7 nrad (0.002 arcsec) of 2D-MNAG and angle monitoring results of Elcomat HR autocollimator.

The 2D-MNAG is incremented in steps of 9.7 nrad (0.002 arcsec) along the *x*-axis, and the output is monitored using both the capacitive-sensor-based angle monitoring unit and the Elcomat HR, as shown in Figure 8. It can be seen that the 2D-MNAG can reliably output increments of 9.7 nrad (0.002 arcsec) along both the *x*-and *y*-axes.

### 4.2. Angle Positioning Repeatability and Output Deviation

According to the simulation results shown in Section 3.2, no suitable instrument is available for determining the 2D-MNAG accuracy. For instance, the accuracy of the typical Elcomat 3000 autocollimator is ±1.5 μrad (±0.3 arcsec) in the range of ±5090 μrad (±1050 arcsec); this is considerably lower than the theoretical angle output accuracy of the 2D-MNAG. Although the Elcomat HR has a suitable accuracy, its measurement range of 727 μrad (±150 arcsec) is considerably lower than the angle output range of ±4363 μrad (±900 arcsec) of the 2D-MNAG. The angle positioning repeatability of the 2D-MNAG is the most important parameter in regards to an evaluation of its working performance.

The angle positioning repeatability of the 2D-MNAG is tested using the setup shown in Figure 7, at angle positions of (0, 0), (0, 300), (0, 900), (600, 600), and (300, 300) arcsec. The measurement range of the Elcomat HR is ±727 μrad (±150 arcsec), which cannot cover all of the above angle positions. Therefore, the auxiliary mirror shown in Figure 7 requires the adjustment and fixing at the Elcomat HR measurement range for each angle position of the 2D-MNAG. Then, to evaluate its angle output repeatability, the 2D-MNAG is moved to different angle positions 20 times.

**Figure 9:**
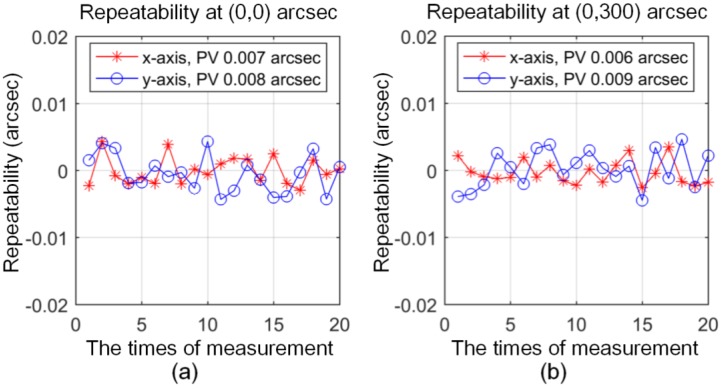

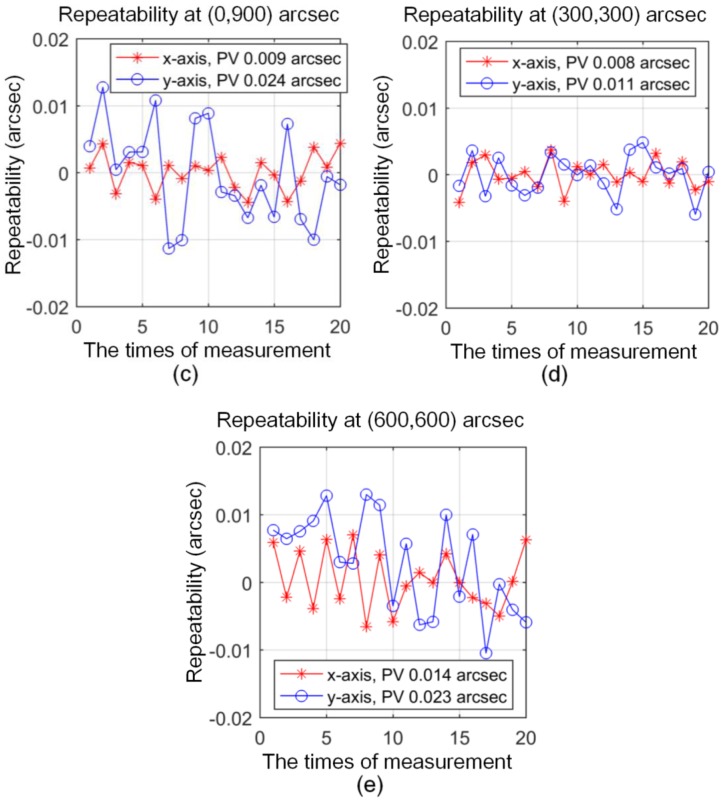
Repeatability test results of 2D-MNAG at: (**a**) (0, 0); (**b**) (0, 300); (**c**) (0, 900); (**d**) (300, 300); and, (**e**) (600, 600) arcsec.

Figure 9a–e show the repeatability test results. It can be seen that the 2D-MNAG has repeatability values of 116 nrad (0.024 arcsec) in the range of ±4363 μrad (±900 arcsec) and 53 nrad (0.011 arcsec) in the range of ±1454 μrad (±300 arcsec) along both the *x*-and *y*-axes; this is suitable for most calibration applications of 2D angle measurement instruments, such as autocollimators.

### 4.3. Scale Factor and Output Deviation Test

**Figure 10:**
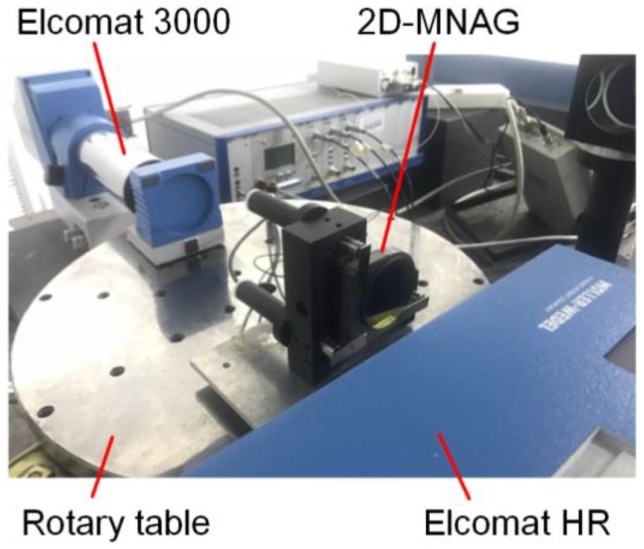
Experimental setup for scale factor and output deviation test of 2D-MNAG.

**Figure 11:**
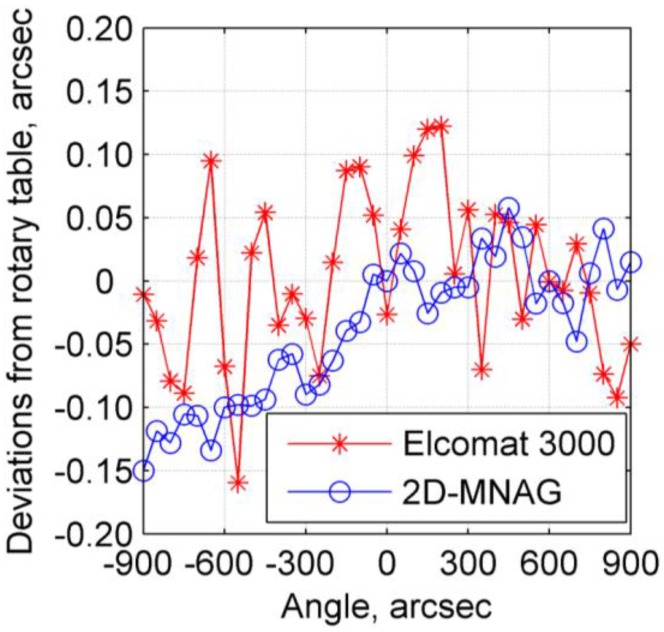
Test results for 2D-MNAG scale factor.

As discussed in Section 4.2, no suitable instrument is available for accuracy determination for the full 2D output range of the 2D-MNAG. However, the 2D-MNAG scale factor can be calibrated using a 1D angle comparator or measurement instrument with high accuracy and a large range in one dimension. The experimental setup for scale factor calibration is shown in Figure 10. The 2D-MNAG scale factor is calibrated using a self-made air-bearing precision rotary table. The circular grating used in the rotary table is Heidenhain RON 886 with an accuracy of ±1 arcsec. The autocollimator Elcomat HR is used as a null-indicating instrument. The 2D-MNAG output is varied with rotation of the rotary table to maintain the Elcomat HR read out at (0, 0) arcsec. In addition, an Elcomat 3000 autocollimator is used to measure the rotation angle of the rotary table for comparison with the output angle given by the rotary table itself. Thus, the scale factor of the 2D-MNAG is well calibrated. The outputs of both the 2D-MNAG and Elcomat 3000 are compared with the data for the rotary table to eliminate impact of relative low accuracy of the rotary table. The deviations are shown in Figure 11, indicating a satisfactory nonlinear scale factor for the 2D-MNAG with a maximum deviation of −0.151 arcsec in the range of ±900 arcsec.

**Figure 12:**
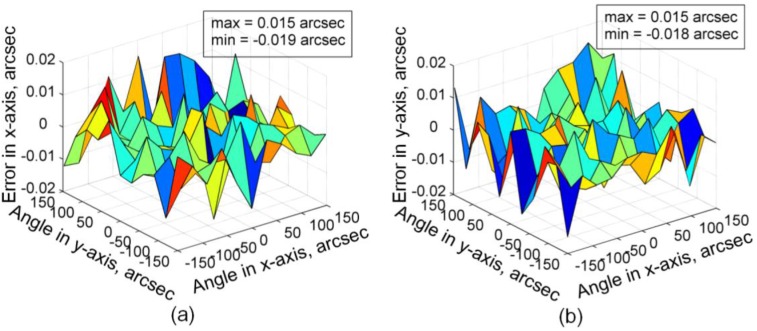
Output deviation of 2D-MNAG when compared to Elcomat HR: (**a**) in *x*-axis; (**b**) in *y*-axis.

The angle output deviation of the 2D-MNAG is also tested within the measurement range of ±150 arcsec of the Elcomat HR with an angle interval of 30 arcsec in 2D, using the setup shown in Figure 10. The test results are shown in Figure 12. It can be seen that the maximum output deviations on the *x*- and *y*-axes are within the range of −0.019 to 0.015 arcsec and −0.018 to 0.015 arcsec, respectively.

## 5. Conclusions

This study has presented a 2D-MNAG that uses a piezo-driven double-axis flexure hinge for 2D angular deflection and three capacitive sensors for 2D output angle monitoring. The principal error of the capacitive sensor for precision microangle measurement was analyzed and compensated for, so as to achieve a high angle output resolution of 9.7 nrad (0.002 arcsec) and positioning repeatability of 116 nrad (0.024 arcsec) over a large angular range of ±4363 μrad (±900 arcsec) for the 2D-MNAG. The expected accuracies of the 2D-MNAG along the *x*- and *y*-axes are 0.264 and 0.319 arcsec in the range of ±900 arcsec, respectively, and 0.082 and 0.098 arcsec in the range of ±300 arcsec, respectively. The impact of each error component together with the synthetic error of the 2D-MNAG after principal error compensation were also determined using Monte Carlo simulation for further improvement of the 2D-MNAG.

## Figures and Tables

**Table 1:** Error sources and their impacts on micro-angle measurement results.

Error source	Distribution Pattern	Distribution Range	Impact at −4363 μrad (−900 arcsec)
Orientation error of normal vector of baseboard on *x*- and *y*-axes			
*α_B_*	Rectangular	±145 μrad (±30 arcsec)	240 nrad (0.0494 arcsec)
*β_B_*	Rectangular	±145 μrad (±30 arcsec)	2 nrad (0.0004 arcsec)
Positioning error of center of sensor plane on *x*-, *y*- and *z*-axes			
*p_x_*	Rectangular	±0.03 mm	759.2 nrad (0.1566 arcsec)
*p_y_*	Rectangular	±0.03 mm	0 nrad (0 arcsec)
Orientation error of normal vector of sensor plane on *x*- and *y*-axes			
*α_C_*	Rectangular	±145 μrad (±30 arcsec)	238 nrad (0.0491 arcsec)
*β_C_*	Rectangular	±145 μrad (±30 arcsec)	2 nrad (0.0004 arcsec)
Distance measurement error of capacitive sensor after calibration			
*d_C_*	Rectangular	10 nm	81.9 nrad (0.0169 arcsec)
Synthetic error			846.0 nrad (0.1745 arcsec)

## Appendix A

As shown in Figure 3, the area of the infinitesimal can be calculated using its width *dx* and distance *x* to the center of the sensor plane, as shown in Equation (A1).

(A1) $$dS=2\cdot \sqrt{R^{2}-x^{2}}\cdot dx$$

The electric field intensity *dE* between the infinitesimal and the baseboard is given in Equation (A2), where *dQ* is the assumed quantity of electric charge on the infinitesimal.

(A2) $$dE=\frac{d\sigma }{\varepsilon_{0}\varepsilon_{r}}=\frac{dQ}{\varepsilon_{0}\varepsilon_{r}dS}$$

The electronic field line can be assumed to be circular arc so that it can be perpendicular to both the sensor plane and the baseboard while they are not parallel to each other. The circular arc length can be calculated using Equation (A3).

(A3) $$l=\left(\frac{d}{\tan\theta }+x\right)\cdot \theta$$

The voltage *dU* between the infinitesimal and the baseboard can be calculated using the electronic field intensity *dE* and the circular arc length *l* of the electronic field line, as shown in Equation (A4).

(A4) $$dU=l\cdot dE=\left(\frac{d}{\tan\theta }+x\right)\theta \cdot \frac{dQ}{2\varepsilon_{0}\varepsilon_{r}\sqrt{R^{2}-x^{2}}dx}$$

The capacitance between the infinitesimal and the baseboard can be calculated using the quantity of electric charge *dQ* and voltage *dU*, as shown in Equation (A5).

(A5) $$dC=\frac{dQ}{dU}=\frac{2\varepsilon_{0}\varepsilon_{r}\sqrt{R^{2}-x^{2}}}{\left(\frac{d}{\tan\theta }+x\right)\theta }dx$$

Then, the capacitance between the sensor plane and the baseboard can be calculated using the integration shown in Equation (A6).

(A6) $$C=\int dC=\int_{-R}^{R}\frac{2\varepsilon_{0}\varepsilon_{r}\sqrt{R^{2}-x^{2}}}{\left(\frac{d+d_{0}}{\tan\theta }+x\right)\theta }dx$$

## Appendix B

The computational model of the capacitive-sensor-based angle monitoring unit is established accurately by using spatial geometrics. The equations of the sensor plane and baseboard are given by Equations (A7) and (A8), respectively.

(A7) $$A\left(x-x_{C}\right)+B\left(y-y_{C}\right)+C\left(z-z_{C}\right)=0$$

(A8) $$m\left(x-x_{B}\right)+n\left(y-y_{B}\right)+p\left(z-z_{B}\right)=0$$

where *P_C_*(*x_C_*, *y_C_*, *z_C_*) is one point and *V_C_*(*A*, *B*, *C*) is the normal vector of the sensor plane, and *P_B_*(*x_B_*, *y_B_*, *z_B_*) is one point and *V_B_*(*m*, *n*, *p*) is the normal vector of the baseboard.

The rotation of the baseboard can be described as the rotation of the point *P_B_*(*x_B_*, *y_B_*, *z_B_*) and the normal vector of *V_B_*(*m*, *n*, *p*) relative to the rotation axis. The rotation matrix is given by Equation (A9).

(A9) $$R=\left[\begin{array}{ccc} C+A_{x}^{2}\left(1-C\right) & A_{x}A_{y}\left(1-C\right)+A_{z}S & A_{x}A_{z}\left(1-C\right)-A_{y}S\\ A_{x}A_{y}\left(1-C\right)-A_{z}S & C+A_{y}^{2}\left(1-C\right) & A_{y}A_{z}\left(1-C\right)+A_{x}S\\ A_{x}A_{z}\left(1-C\right)+A_{y}S & A_{y}A_{z}\left(1-C\right)-A_{x}S & C+A_{z}^{2}\left(1-C\right) \end{array}\right]$$

where *C* = cos*θ*, *S* = sin*θ*, *θ* is the rotation angle, and (*A_x_*, *A_y_*, *A_z_*) is the normalized direction vector of the rotation axis.

The point PBR and normal vector VBR after rotation can be calculated using Equation (A10).

(A10) $$P_{BR}=R\cdot P_{B},\;V_{BR}=R\cdot V_{B}$$

The intersection point of the axis of the capacitive sensor and the plane equation of the baseboard can be calculated using Equation (A11). The distance from the intersection point to the center of the sensor plane can be calculated using Equation (A12), which is the actual distance d from the center of the sensor plane to the baseboard.

(A11) $$\left[\begin{array}{l} x_{I}\\ y_{I}\\ z_{I} \end{array}\right]=\frac{1}{mA+nB+pC}\left[\begin{array}{l} mAx_{C}+\left(nB+pC\right)x_{B}+mB\left(y_{C}-y_{B}\right)+mC\left(z_{C}-z_{B}\right)\\ nA\left(x_{C}-x_{B}\right)+nBy_{C}+\left(mA+pC\right)y_{B}+nC\left(z_{C}-z_{B}\right)\\ pA\left(x_{C}-x_{B}\right)+pB\left(y_{C}-y_{B}\right)+pCz_{C}+\left(mA+nB\right)z_{B} \end{array}\right]$$

(A12) $$d=\sqrt{{\left(x_{C}-x_{I}\right)}^{2}+{\left(y_{C}-y_{I}\right)}^{2}+{\left(z_{C}-z_{I}\right)}^{2}}$$

The angle *θ* between the sensor plane and the baseboard is determined by their normal vectors, as shown in Equation (A13). The angle *θ* is used in Equations (6)–(8) in Section 3.1 to calculate the principal error of the capacitive-sensor-based angle monitoring unit for further compensation of the angle measurement results.

(A13) $$\theta =\arccos\left(\frac{Am+Bn+Cp}{\sqrt{A^{2}+B^{2}+C^{2}}\sqrt{m^{2}+n^{2}+p^{2}}}\right)$$
